# Diversity and selection of MHC class I genes in the vulnerable Chinese egret (*Egretta eulophotes*)

**DOI:** 10.1371/journal.pone.0176671

**Published:** 2017-05-03

**Authors:** Zeng Wang, Xiaoping Zhou, Qingxian Lin, Wenzhen Fang, Xiaolin Chen

**Affiliations:** Key Laboratory of the Ministry of Education for Coastal and Wetland Ecosystems, College of the Environment and Ecology, Xiamen University, Xiamen, Fujian, People's Republic of China; National Cheng Kung University, TAIWAN

## Abstract

The genes of major histocompatibility complex (MHC) are important to vertebrate immune system. In this study, two new MHC class I genes, designated as Egeu-UAA and Egeu-UBA, were discovered in the vulnerable Chinese egret (*Egretta eulophotes*). Using a full length DNA and cDNA produced by PCR and RACE methods, these two MHC class I loci were characterized in the genome of the Chinese egret and were also found to be expressed in liver and blood. Both new genes showed the expected eight exons and were similar to two copies of the minimal essential MHC complex of chicken. In genetic diversity, 14 alleles (8 for UAA and 6 for UBA) in the MHC class I gene exon 3 were found in 60 individuals using locus-specific primers and showed little polymorphism. Only three potential amino acid residues were detected under positive selection in potential peptide-binding regions (PBRs) by Bayesian analysis. These new results provide the fundamental basis for further studies to elucidate the molecular mechanisms and significance of MHC molecular adaptation in vulnerable Chinese egret and other ardeids, finding that have not been previously reported.

## Introduction

The major histocompatibility complex (MHC) is a gene complex that encodes for cell-surface proteins responsible for the recognition and presentation of foreign antigens to T-lymphocytes, and which triggers the adaptive branch of the immune system [1]. The MHC molecules can be divided into three classes, class I, class II and class III [2]. Class I and class II molecules have similar folding patterns, differ in their domain organization and are at best as similar in sequence to each other as to many other molecules. Class II molecules are involved in the adaptive immune response, but class I is recognised by T cells of the adaptive immune system and NK cells of the innate immune system. Some genes in the class III region encode molecules involved in lymphocyte interactions or with innate immunity. MHC class I molecules function to present peptides that arise from proteins expressed in the cytoplasm to contiguous organelles including the nucleus (often derived from what are referred to as intracellular pathogens). In contrast, MHC class II molecules present peptides that arise in intracellular vesicles and extracellular space (often derived from what are referred to as extracellular pathogens). Since each MHC molecule can successfully bind a limited number of peptides, greater polymorphism at the peptide-binding regions (PBRs) should increase the number of pathogens that can be recognized by an individual, resulting in balancing selection for disease resistance and genetic diversity [3, 4].

The MHC is known to be a complicated gene group in vertebrates, both in terms of allelic diversity and gene number [5]. Numerous studies have suggested that selection for resistance and immunity from parasites and disease and possibly sexual selection are the major factors driving this polymorphism [6–8]. Thus, several studies have emphasized the potential of MHC genes as valuable molecular markers to assess the evolutionary and adaptive potential of endangered populations and species in relation to the menace of changed and emerging diseases [9–12].

Despite continuing investigation of avian MHC class I genes, and an increasing interest in their use as markers of adaptive genetic variation in evolutionary ecology research [13–15], many important gaps in our knowledge of MHC class I multigene family evolution in birds remain largely unexplored. Many studies have focused upon the isolation of cDNA transcripts or the variation within the polymorphic PBR exons, with the result that no full-length sequences have been described for non-galloanseriforms. However, the limited DNA sequence information, including differences in gene copy number, the presence of pseudogenes, and the inability to identify orthologous loci from sequence similarity in other unrelated species makes characterization of the MHC class I genes in more species of fundamental importance. For ornithological studies, locus-specific amplification is commonly hampered by the existence of highly conserved sequences surrounding the PBR, and locus assignment based upon PBR sequence similarity is often impeded by conflicting evolutionary signals due to recent duplication, recombination and/or concerted evolution [16]. The evolutionary forces acting upon each locus cannot be untangled from the larger issue of polymorphism and selective pressures averaged across all loci. Similarly, the relative contributions of classical versus nonclassical genes to the overall variation cannot be adequately evaluated. For these reasons, our current knowledge of avian MHC class I genes remains taxonomically restricted, which limits the potential of comparative methods and a broader understanding of the evolutionary processes acting upon these genes.

The Chinese egret (Ardeidae, *Egretta eulophotes*) is listed as a vulnerable species with an estimated global population of 2600–3400 individuals [17]. This egret is a migratory colonial waterbird, wintering in the south of Asia and breeding on offshore islands in Russia, North Korea, South Korea and China. The migratory colonial patterns of this egret may drive MHC polymorphisms as migratory birds would inevitably face exposure to a more diverse fauna of pathogens and parasites, and colonial birds would elevate transmission rates of pathogens [18]. Previous studies of this species have focused on mitochondria [19], microsatellite [20] and MHC class II genes [21–23]. However, up to the present, there are no studies on MHC class I genes in any heron or egret species, which is the first step towards understanding the role of pathogen-mediated selection and mate choice in the maintenance of MHC class I diversity. Specifically, this study aims to: (1) isolate full genomic and cDNA MHC class I sequences from the Chinese egret (*Egretta eulophotes*), (2) design locus-specific primers to survey the variation in PBR-encoding exons for all loci and (3) gain insights into the selection pressures acting on PBR and non-PBR regions within the third exon of MHC class I loci in the Chinese egret.

## Materials and methods

### Ethics statement

This research and all procedures involving collection of animal tissue in the wild were approved by the Administration Center for Wildlife Conservation in Fujian Province (FJWCA-1208). The scientific license for access to the study site was issued by the Administration Department of Xiamen Egret Natural Reserve (XMENR-1005). Blood samples (~0.5mL) were collected from 60 nestlings (aged around 15 days) of *E*. *eulophotes* by puncturing the wing vein and using a syringe to withdraw the blood. The nestlings were immediately returned to the nest after staunching the wound with cotton. Collection of the blood samples was conducted during the morning, and visits to the breeding colony were restricted to a maximum of two hours per day.

### Methods for DNA extraction, RNA extraction and reverse transcription

Blood samples of 60 nestlings were collected from 35 nests at Riyu Islet (27°01′N, 120°25′E) in Fujian Province of China, where a total of 50 nests of the Chinese egret were found. The blood samples were frozen at −80°C until DNA extraction. Total genomic DNA was isolated using the Universal Genomic DNA Extraction Kit Ver. 3.0 (TaKaRa), and total RNA was extracted using Trizol (Invitrogen, Switzerland) from the blood and liver of one individual Chinese egret that died at this Islet, respectively (S1 Fig). One μg of total RNA was then reverse transcribed into first-strand cDNA with PrimeScript RT Master Mix (TaKaRa), according to the manufacturer’s instructions.

### Isolation of MHC class I genes

A fragment from exon 2 to exon 4 of MHC class I genes in Chinese egret was first amplified with degenerate primers MHCI-ex2F: CGCTACAACCAGASCRRSG and MHCI-ex4R: GGGTAGAAGCCGTGAGCRC [24]. PCR reactions were carried out on a Biometra T gradient thermocycler in a final volume of 20 μL, which containing 1 × PCR buffer (50 mM KCl, 10 mM Tris-HCl, pH 8.3, 1.5 mM MgCl_2_), 0.2 mM of each dNTP, 0.4 μM of each primer, 0.7 U of Taq polymerase (TaKaRa), 100 ng of genomic DNA. The conditions for PCR amplification were a denaturing step at 94°C for 3 min, followed by 35 cycles at 94°C for 30 s, 55°C for 30 s, 72°C for 2 min, and a final extension at 72°C for 10 min.

The 3’ and 5’ rapid amplification strategy of cDNA ends (RACE) [25] was used to isolate an entire coding sequence and the gene transcription was confirmed by using 3’-full RACE core set ver. 2.0 (TaKaRa) and 5’-full RACE Kit (TaKaRa). Based on the DNA sequence obtained from the fragment, gene specific outer/inner primers were designed in conserved exon 4 regions for 3’ RACE (13sp1: CGTGGTCCGAGTGTCG 13sp2: GACCCTGACCTTGTCCTGC) and 5’ RACE (15sp1: GGTACTTGTCCTTCTCCTCCG 15sp2: CCAGGTGTAGTAGGTGCCATC) respectively, using Oligo 6.0 (Molecular Biology Insights). PCR reactions followed the protocols and applications as specified in the kit. A 730bp and an 810bp segments were obtained from 3’ RACE and 5’ RACE, respectively. After removing their overlapping region, a 1380bp long intact gene fragment of MHC class I was obtained.

According to the complete cDNA sequence, two primers, 15utrF CAGAACTCTGCCCGGAGACGG and 13utrR AGCGTGCACAGGGAGCAGAAATCAG were designed to amplify the complete DNA sequence of the MHC class I gene. PCR reactions were carried out on a Biometra T gradient thermocycler in a final volume of 50 μL containing 1× LA PCR buffer II, 2.5 mM MgCl_2_, 0.4 mM dNTP, 0.5 μM of each primer, and 2 U TaKaRa, LA Taq. PCR conditions included an initial denaturation step at 95°C for 2 min, 35 cycles of denaturation at 98°C for 10 s, extension at 68°C for 15 min, and a final extension at 72°C for 10 min.

All PCR products were purified using the Agarose Gel DNA Purification Kit Ver. 2.0 (TaKaRa), then ligated into pMD 18-T vector (TaKaRa) and transformed into *Escherichia coli* DH5α. The genes of over 3000 bp were sequenced by using Primer Walking method from both sides respectively. In this Primer Walking method, after a shorter stretch (about 800bp) had been sequenced using M13F or M13R, a new primer was generated from the end of what had been already sequenced and the process was continued until the sequence was overlapped. Ten positive colonies of each band were selected to sequence bidirectionally on an automatic sequencer (ABI PRISM 3730; Invitrogen Biotechnology Co. Ltd.) using universal M13 sequencing primers and BigDye version 3 (Applied Biosystems).

### Polymorphism of the MHC class I genes complex

To detect polymorphism within the MHC class I genes in wild populations of the Chinese egret, 60 nestlings on the Riyu Islet were genotyped by semi-nested PCR combined with single strand conformation polymorphism (SSCP). First, two forward locus-specific primers in intron 2 were designed and designated as UAAF and UBAF (UAAF: CATCCCCGTGGCAGTCGAGA and UBAF: AATCCCATCTCCTTGTCCCATCTTCC). These were combined with the reverse primer 13exR: CGTAGCTCACGTATTTCCTCAGCCAC to amplify the two loci, respectively. PCR was carried out in a 20 μL reaction mixture containing 1 μL genomic DNA, 0.7 U of Taq polymerase (TaKaRa), 1.5 mM MgCl_2_, 200 μM of each dNTP and 0.4 μM of each primer, for 25 cycles at 94°C for 30 s, 60°C for 30 s and 72°C for 1min. Second, to obtain suitable length fragments for SSCP genotyping and scanning of the variation in exon 3 of each locus, a second round PCR was carried out using the primer 13exF: ACTGAAGGCCCAGGGCTGC and 13exR was used to amplify the entire exon 3 in each locus. The reaction conditions for the second round PCR were identical with those described for the first round. The PCR products from the first round were diluted 100-fold and used as the template. The amplicons were separated by SSCP analyses as described by Fain [26]. Five μL of each PCR product were mixed with an equal volume of 95% formamide, denatured for 5 min at 99°C and immediately chilled on ice water for 10 min. The reaction mixture was loaded on an 8% nondenaturing polyacrylamide gel (PAGE) (37.5:1). Electrophoresis was carried out at 10°C for 5 h at 8 W per gel followed by a sensitive silver-staining procedure, as described by Budowle [27]. Samples with equal banding patterns were rearranged and electrophoresed a second time on a nondenaturing PAGE on adjacent lanes to ensure the genotyping, and samples with unique genotypes were typed using two independent PCR reactions. All SSCP-bands were cut from at least two individuals of each genotype and incubated in 80 μL water for 3 h at 37°C. One μL was used as template in a second PCR under the same conditions as described above. The products were screened in an additional SSCP analysis and every allele was directly sequenced in both directions from at least two individuals or two independent PCRs from one individual.

### Exclusion of PCR errors and definition of alleles

For all PCR reactions, the amplification, cloning and sequencing was carried out twice and only sequences found in both experiments were included in the analyses to avoid the inclusion of PCR artifacts. Since recombination of cloned PCR products can introduce additional artifacts [28, 29], direct sequencing of uncloned PCR products was used for agreement with the polymorphic sites. The bands excised from SSCP were directly sequenced in both directions. Only completely identical sequences found in two independent PCR events were defined as alleles. These alleles are referred to as “verified” because the same sequence is highly unlikely to arise twice from independent amplification errors. Throughout this study, the word “allele” is used to describe a 273 bp exon 3 sequence derived from SSCP genotyping.

### Data analyses

The complete DNA and cDNA sequences for MHC class I genes, derived from the Chinese egret, and were aligned using the DNAMAN software package (Lynnon Biosoft, Quebec, Canada). Exons and introns were distinguished using the GenScan Program [30]. DNA fragments obtained from the 60 nestlings were edited in the BioEdit Sequence Alignment Editor [31]. Alignment gaps were treated as missing data in all analyses. Sequence variability statistics were calculated using the Mega 4.0 Program [32]. The proportion of synonymous (d_S_) and non-synonymous (d_N_) substitutions were calculated for both the 18 possible PBRs as defined by Bjorkman and Saper [33, 34], and the remaining non-antigen binding sites (non-PBRs) for each of the two loci. The Z-Test in the Mega 4.0 was used to test for directed selection. Standard errors were obtained with 1,000 bootstrap replicates, including average rates of synonymous (d_S_) and nonsynonymous (d_N_) substitutions per site according to the Nei–Gojobori method with Jukes–Cantor correction for multiple substitutions [35]. Population allele frequencies and tests of deviation from Hardy–Weinberg equilibrium were calculated using GENEPOP 4.0 [36]. The programme CODEML in the PAML package, version 3.15 [37] was used to test for the presence of codon sites affected by positive selection and to identify those sites in exon 3 sequences of the Chinese egret, and the neutral model M7 and the positive selection model M8 were compared using maximum likelihood ratio tests (LRT).

## Results

### Characterization of MHC class I genes

**Fig 1:**
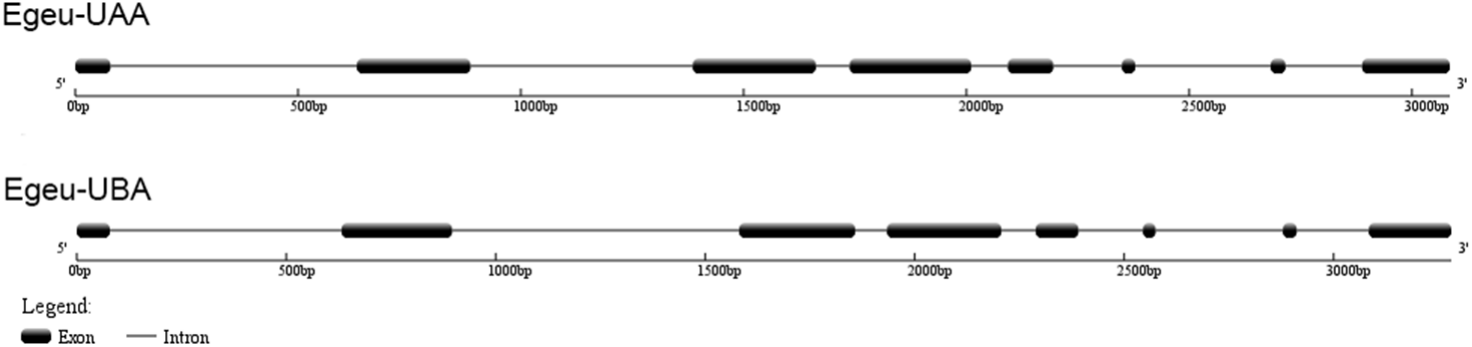
Schematic illustration of MHC class I genes in the Chinese egret.

**Fig 2:**
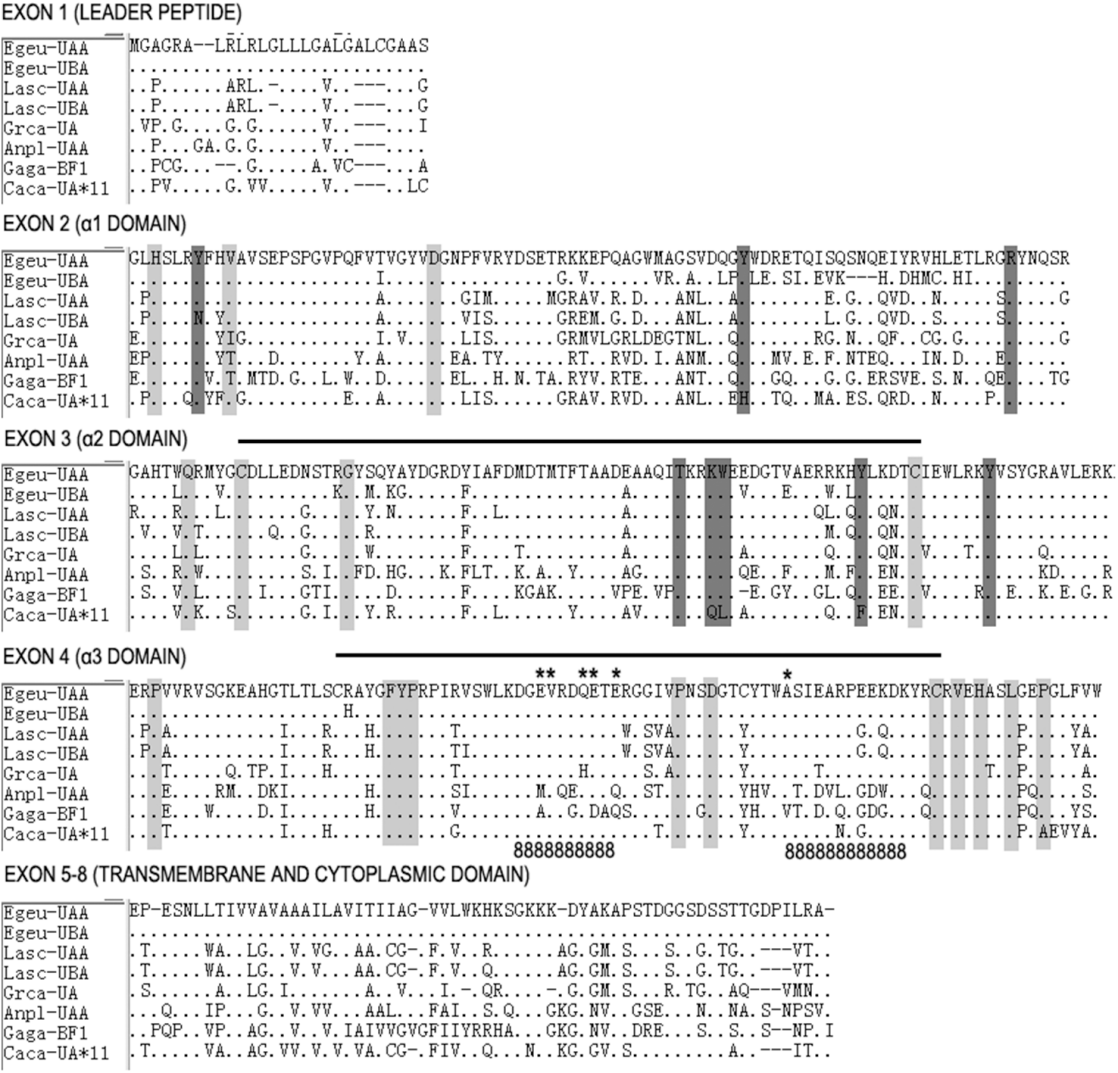
Comparison of amino acid alignment of transcribed MHC class I alpha chain sequences in the Chinese egret with those in other bird species. *Dots* indicate identity with Egeu-UAA, *dashes* indicate gaps. Conserved sequence features with predicted functional or structural roles are shown as: peptide main chain binding residues (*dark grey shading*), intra- and inter-domain contacts (*light grey shading*), intra-domain disulfide bridges (*horizontal lines*), CD8 binding sites (*“8”s*), critical CD8 co-receptor sites (*) [33, 34, 39, 40]. Sequence sources are: Grca (Florida sandhill crane, *Grus canadensis pratensis*, AF033106); Gaga (chicken, *G*. *gallus* BF2, AL023516); Anpl (mallard duck, *Anus platyrhynchos* UAA, AY885227); Lasc-UAA (red-billed gulls, *Larus scopulinus* UAA, HM015819); Lasc-UBA (red-billed gulls, *Larus scopulinus* UBA, HM015820); Caca (Red Knot, *Calidris canutus*, KC205116).

In this study, DNA sequences of two different MHC class I genes were isolated from one individual Chinese egret, and the sequences were highly similar over large stretches. They could be distinguished, however, by the divergent intron 2. The exon sizes were similar to those found in other birds (Fig 1, S2 Fig). According to Klein [38], these two genes were designated as Egeu-UAA and Egeu-UBA (GenBank Accession KY511591- KY511592). The result of RACE analyses indicated that each of the two genes comprised a total of 1080 bp encoding for 360 amino acid residues in the exon sequences. These encoded for eight of the expected exons including those encoding the signal peptide, the alpha 1 (α1), alpha 2 (α2) and alpha 3 (α3) domains, the transmembrane region, and the cytoplasmic tail (Fig 2). No frame shifts or premature stop codons were observed, but the canonical donor–acceptor splice sites (GT/AG) and polyadenylation recognition sequences (AATAAA or ATTAAA) were present in all sequences.

### Sequence variation in exon 3

**Fig 3:**
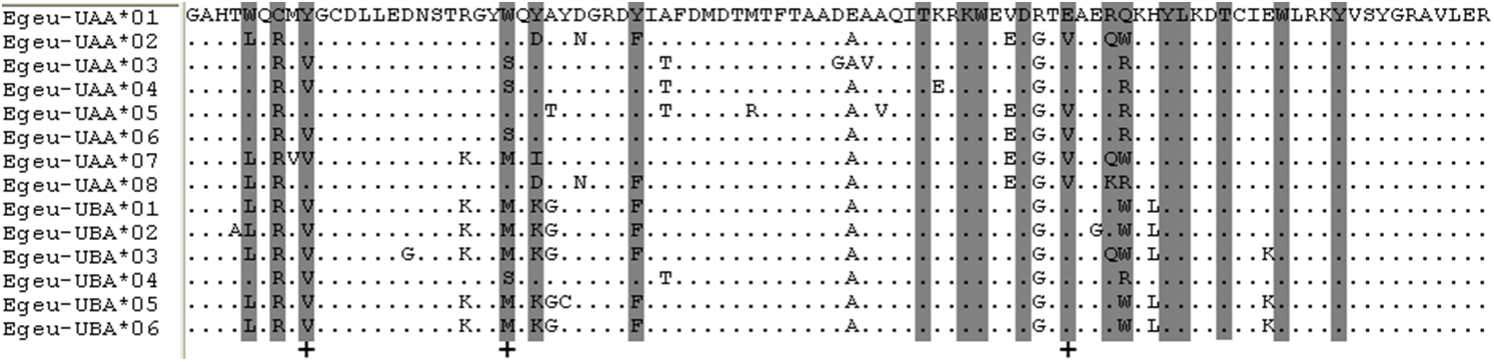
Alignment of translated amino acid sequences for exon 3 of MHC class I genes in the Chinese egret. A total of 14 alleles from two loci are shown. *Dots* indicate identity to the majority rule consensus sequence. Codons corresponding to PBR residues in human HLA-A2 are *shaded* [33, 34]. The presumed sites that positive selection is acting upon are indicated by *plus sign* (on alignment bottom).

Based on the Egeu-UAA and Egeu-UBA sequences, two forward locus-specific primers were designed on intron 2 for each locus, respectively. These two sequences were then combined with the universal primers to amplify the exon 3, as described above. Single locus genotyping of MHC class I was carried out on 60 nestlings. The SSCP results showed one or two alleles per individual at each locus (S3 Fig, S1 Table), confirming the presence of two loci. Fourteen alleles in total were obtained from the two loci, including 8 from Egeu-UAA and 6 from Egeu-UBA (GenBank Accession KY511593- KY511606). Translation of the nucleotide sequences produced 14 unique amino acid sequences (Fig 3).

### Frequency of allele and Hardy-Weinberg equilibrium

In this study, our sample size was appropriate for the analyses of allele frequency and Hardy-Weinberg Equilibrium because 60 samples were sufficient to represent the full genetic variation present in this islet population. According to the optimal sampling strategy of Marshall et al [41] and Sjögren et al [42], approximately 30 individuals from a finite population could be considered as a recommended size for collection in genetic studies. Our allele frequency results (Table 1) showed that alleles of each locus had an uneven distribution. At each locus, some alleles had a higher frequency than others, such as Egeu-UAA*02, Egeu-UAA*04, Egeu-UBA*01 and Egeu-UBA*04. The departure from Hardy-Weinberg equilibrium within each locus was statistically significant (P<0.001).

**Table 1:** Allele frequency of MHC class I genes in the Chinese egret.

Allele\ Locus	Egeu-UAA	Egeu-UBA
01	0.0583	0.2750
02	0.3833	0.0417
03	0.0583	0.1167
04	0.2833	0.4583
05	0.0500	0.0917
06	0.0833	0.0167
07	0.0500	
08	0.0333	

### Tests of selection

When the ratio of non-synonymous to synonymous substitution (d_N_/d_S_) was analyzed in predicted peptide-binding region (PBR) sites, and non-PBR sites for each gene separately [43], signs of positive selection were only found in the PBR sites of the two loci, with a d_N_/d_S_ ratio of 1.23 and 1.30 respectively. The ratios were not significantly different than 1, indicating that strong selection was not acting on these two loci in the Chinese egret (Table 2). Furthermore, we tested if positive selection was acting on the exon 3 using CODEML. The LRT values comparing M7 and M8 models indicated that M8 fitted the data significantly better than M7 (P<0.001) in the Egeu-UAA locus. The M8 model identified positive section at the Egeu-UAA locus in which there were three identified codons, 9, 23, and 62 (>95% posterior probability) which corresponded to the potential peptide binding codons identified in human HLA-A2 (Fig 3). As shown in Table 3, the Egeu-UBA locus showed no evidence of positive selection for the LRT (2.1824), indicating that the M8 model did not fit better than M7 (P>0.1). Additionally, no amino acid sites in exon 3 were detected under strong positive selection (confidence interval level is greater than 95%).

**Table 2:** Summary of sequence variation of MHC class I in the Chinese egret.

	S_nt_	S_AA_	π	d_N_	d_S_	d_N_/d_S_	p	z
PBR site								
Egeu-UAA	21	9	0.226±0.052	0.237±0.085	0.192±0.098	1.23	0.325	0.454
Egeu-UBA	11	6	0.076±0.024	0.082±0.036	0.063±0.045	1.30	0.063	1.544
Non-PBR site								
Egeu-UAA	19	13	0.029±0.007	0.024±0.007	0.046±0.019	0.52	1	-1.081
Egeu-UBA	15	9	0.026±0.006	0.020±0.006	0.046±0.019	0.43	1	-1.340

Total size 273bp (91 residues) for all sites, 54 bp (18 residues) for PBR site according to Bjorkman [33] and the remaining exon 3 (non-PBR); S, number of variable nucleotide (nt) or amino acid (AA) sites; π, nucleotide diversity ± SE; d, average rate of nonsynonymous (d_N_) or synonymous (d_S_) substitutions per site ± SE; z, the Z test of positive selection.

**Table 3:** Summary of parameter estimates and likelihood values of different models of codon evolution for exon 3 of MHC class I genes in the Chinese egret.

Locus	Model	ln L	Parameter estimates	Positively selected sites	2△L(LRT)
UAA	M7	-629.6531	p = 0.12151, q = 0.25261	Not allowed	20.1250 (P<0.001)
	M8	-619.5906	p0 = 0.94647, p = 0.30241, q = 0.69069, (p1 = 0.05353), ω = 12.0695	9Y** 23W** 62E**	
UBA	M7	-488.2672	p = 0.00500, q = 0.00527	Not allowed	2.1824 (P>0.1)
	M8	-487.1760	p0 = 0.87182, p = 0.28231, q = 1.19411, (p1 = 0.12818), ω = 4.72865	5L 23M 25K 65R	

Sites inferred to be under positive selection at the 99% (**) confidence interval level are indicated. lnL, log-likelihood value; ω, selection parameter; p_n_, proportion of sites that falls into ω_n_ site class. The sites consistent with human PBR site are indicated in *bold*.

## Discussion

Using a PCR-based approach, two full-length MHC class I genes in the Chinese egret were isolated and characterized. These two genes were newly discovered in Ardeidae, a non-model avian species. Sequence features of antigen-presenting MHC class I genes were largely conserved in each gene, and no frameshift mutations, premature stop codons, or inappropriate splice signals were observed. Additionally, cDNA sequences isolated for each locus indicated that both of them were transcriptionally active in liver and blood. The MHC class I genomic architecture and complexity in the Chinese egret conformed to previous data collected in the nonpasserine birds [44].

In this study, intron 2 of the MHC class I genes in the Chinese egret was found to be more divergent than other regions, making it a likely candidate for locus specific primers in single locus typing of MHC class I. Further, the SSCP results showed one or two alleles per individual on each locus, conforming that the loci were separate. The low number of loci in this egret would allow us to completely genotype each locus of the MHC class I in individual egrets. Moreover, the primers used in this study were designed for targeting highly conserved regions across class I genes, which will be useful for successfully cross-amplifying similar fragments in other Ardeid species [24, 43, 45].

Sequence features typical of antigen-presenting MHC class I genes in the Chinese egret were largely conserved in both loci. Eight residues in the α1 and α2 domains contacted main chain atoms of the bound ligand, and anchored the peptide termini in a sequence-independent manner (Fig 2, in dark grey shading) [33, 34]. In classical MHC class I genes of human and mouse, no more than two amino acid replacements exist at these sites, with substitutions tending to be of a conserved nature [46, 47]. The Egeu-UAA and Egeu-UBA in the Chinese egret showed no deviation from the consensus “YYRTKWYY” sequence in which arginine is substituted for tyrosine at alignment position 87 in non-mammalian vertebrates [48).

The “Major “class I genes with high sequence variation and high expression levels across tissue types have been described for some bird species such as the chicken, duck, red-billed gull and crested ibis [44, 48–50]. Among the classical I genes of the Chinese egret, genetic diversity in Egeu-UAA locus was higher than the other locus, and the number of alleles obtained from this locus by RT-PCR was also higher. Additionally, the d_N_/d_S_ values (PBR site) suggested that Egeu-UAA locus was under positive selection. These findings suggest that Egeu-UAA represents a major class I gene in the Chinese egret. In contrast to the greater sequence variation of Egeu-UAA, Egeu-UBA showed extremely limited variation and no evidence of positive selection on exon 3, suggesting that this locus may represent a minor MHC class I gene. Moreover, CODEML analyses revealed only 3 residues with posterior probabilities of positive selection (P≥0.95) in Egeu-UAA exon 3, which is a finding significantly difference with most previous research results [44, 51]. This low number of positive selection sites was also found in recent studies of the greater prairie chickens (*Tympanuchus cupido*) [52] and several falcon species [53]. A possible explanation for the lack of both polymorphism and detectable balancing selection at the locus is that strong directional selection has acted on this locus and reduced variation at the PBR. Another alternative explanation for this phenomenon is that drift in this egret population has removed most evidence of selection at this locus. In the future, more studies on other populations of the Chinese egret are needed in order to demonstrate whether drift has erased traces of diversifying selection on the MHC class I loci in this population or strong directional selection has acted on this locus throughout the range of the species.

## Supporting information

S1 Fig Total RNA extracted from the sample and amplification products from cDNA. Total RNA is extracted from the liver and blood. The PCR products are amplified from liver and blood cDNA using primer MHCI-ex2F and MHCI-ex4R. M stands for marker DL2000(Takara).
(TIF)

S2 Fig Sequence alignment of MHC class I loci in crested ibis and red billed gull. The sequences from the MHC class I loci in crested ibis (Nini) and red-billed gull (Lasc) are compared; an mRNA sequence is used to mark the location of the exons (Nini-UCA1M and Lasc-UAAM), respectively. Sequence alignment and the differences between loci in intron 2 from each species are depicted in the genomic plot. The red box is used to mark the position in full length DNA sequence. DNAMAN is used to align the sequences. Sequence sources are: crested Ibis (KP182409 KP182408) and red billed gull (HM008713 HM008714 HM008715 HM008716 HM015819).
(TIF)

S3 Fig SSCP gel image and genotyping results. (TIF)

S1 Table SSCP genotyping result of 60 nestlings from Riyu Islet on UAA and UBA loci. The typing results for the first 15 individuals of the UAA locus are shown in S3 Fig as an illustration.
(XLS)
