# Supplementary figures and images for: Diversity and selection of MHC class I genes in the vulnerable Chinese egret (Egretta eulophotes)
Source: PLoS One. 2017 May 3;12(5):e0176671. doi: 10.1371/journal.pone.0176671 (PMC5415105; doi:10.1371/journal.pone.0176671)

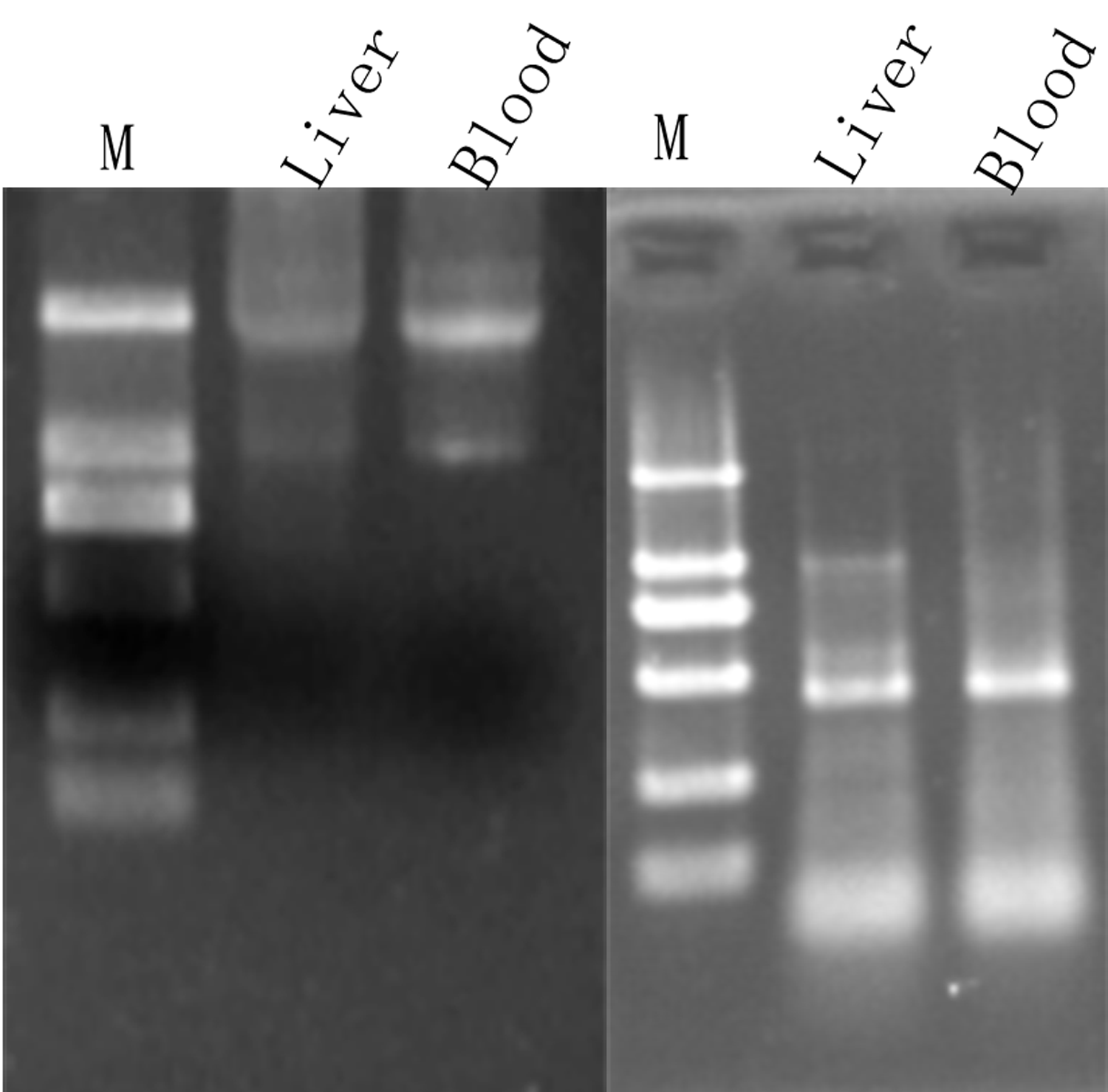

Supplement: S1 Fig — Total RNA is extracted from the liver and blood. The PCR products are amplified from liver and blood cDNA using primer MHCI-ex2F and MHCI-ex4R. M stands for marker DL2000(Takara). (TIF) [file pone.0176671.s001.tif]

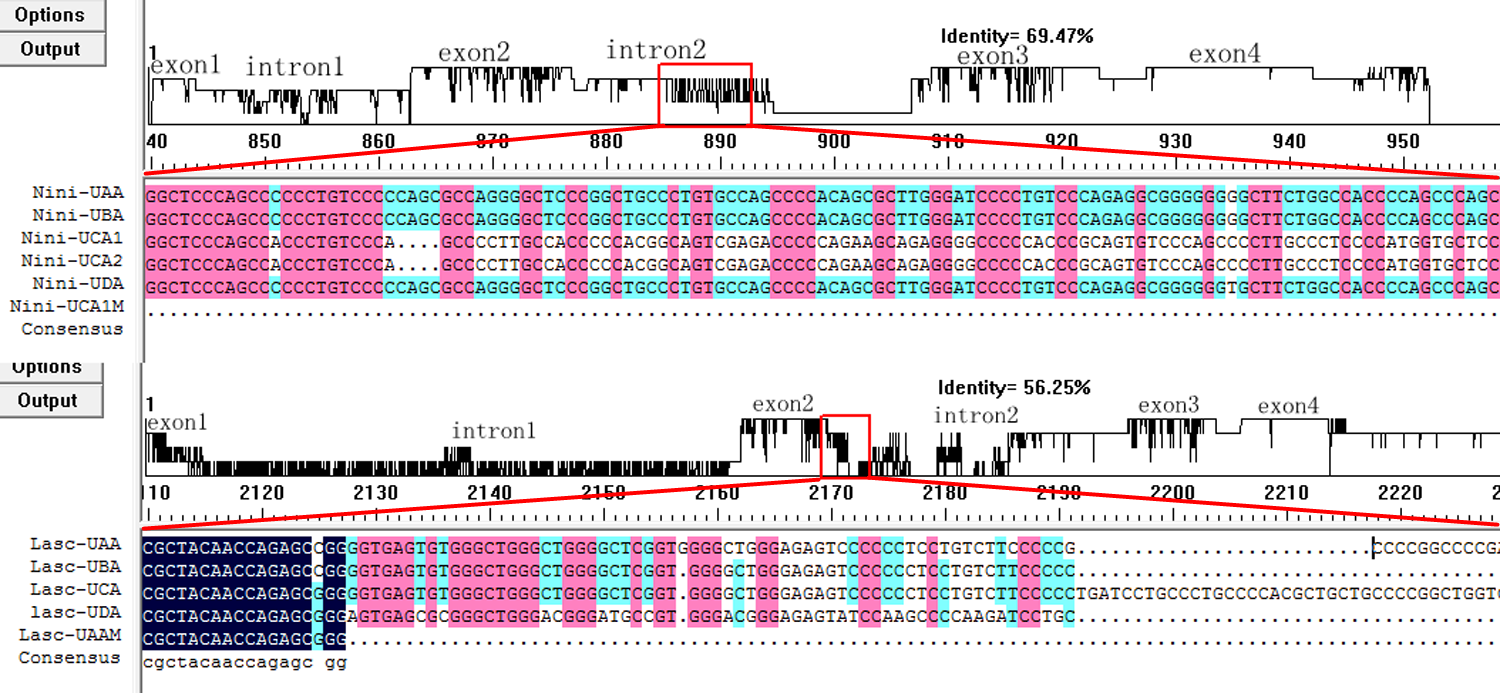

Supplement: S2 Fig — The sequences from the MHC class I loci in crested ibis (Nini) and red-billed gull (Lasc) are compared; an mRNA sequence is used to mark the location of the exons (Nini-UCA1M and Lasc-UAAM), respectively. Sequence alignment and the differences between loci in intron 2 from each species are depicted in the genomic plot. The red box is used to mark the position in full length DNA sequence. DNAMAN is used to align the sequences. Sequence sources are: crested Ibis (KP182409 KP182408) and red billed gull (HM008713 HM008714 HM008715 HM008716 HM015819). (TIF) [file pone.0176671.s002.tif]

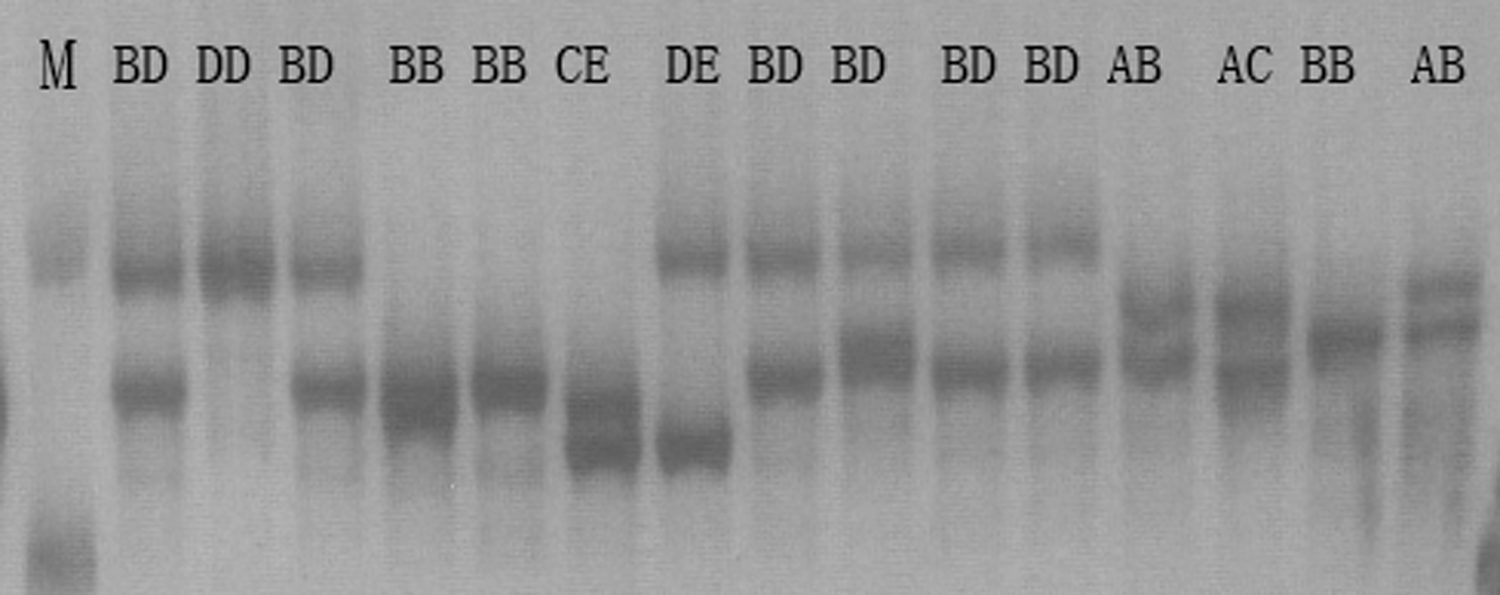

Supplement: S3 Fig — (TIF) [file pone.0176671.s003.tif]
